# Viscoelastic Foams with Enhanced Fire Resistance Using Additive and Reactive Flame Retardants

**DOI:** 10.3390/polym17182459

**Published:** 2025-09-11

**Authors:** Grzegorz Węgrzyk, Dominik Grzęda, Milena Leszczyńska, Anna Bryśkiewicz, Katarzyna Bulanda, Mariusz Oleksy, Joanna Ryszkowska

**Affiliations:** 1 Department of Ceramics and Polymers, Faculty of Materials Science and Engineering, Warsaw University of Technology, PL-02-507 Warsaw, Poland; grzegorz.wegrzyk.dokt@pw.edu.pl (G.W.); dominik.grzeda.dokt@pw.edu.pl (D.G.); aniabryskiewicz@gmail.com (A.B.); joanna.ryszkowska@pw.edu.pl (J.R.); 2 Department of Polymer Composites, Faculty of Chemistry, Rzeszow University of Technology, PL-35-959 Rzeszow, Poland; k.bulanda@prz.edu.pl (K.B.); molek@prz.edu.pl (M.O.)

**Keywords:** viscoelastic foams, flammability, non-halogenated reactive flame retardants

## Abstract

The influence of non-halogenated additive flame retardants, expandable graphite (EG) and ammonium polyphosphate (APP)—as well as a reactive phosphorus-containing polyol, on the flammability, thermal stability, physico-mechanical performance, and morphology of viscoelastic polyurethane foams (VEFs) was investigated. For this purpose, a series of polyurethane foams incorporating both additive and reactive flame retardants was synthesized and analyzed. The incorporation of flame retardants led to a substantial enhancement of fire resistance, as evidenced by an increase in the limiting oxygen index (LOI) to 28–31%, achievement of the UL-94 V_0_ flammability rating, and a 92% reduction in peak heat release rate (pHRR) compared to the unmodified reference foam. Alterations in mechanical performance were correlated with structural changes both at the microscopic and molecular level, confirmed by scanning electron microscopy (SEM), thermogravimetric analysis (TGA), and differential scanning calorimetry (DSC).

## 1. Introduction

Flexible polyurethane foams account for nearly 40% of total PU production. Their widespread use in furniture, mattresses, automotive components, handbags, footwear, and textiles stems from their low density and high elasticity [1,2,3]. The growing demand for PU foams has spurred extensive research aimed at improving their performance. One of the main limitations of these materials is their high flammability, which arises from their porous-often open-cell-structure. This morphology facilitates oxygen diffusion and flame propagation [4]. Additionally, blowing agents such as cyclopentane, n-pentane, methylene chloride, and chlorofluorocarbons, commonly used in PU foam production, further contribute to their flammability [5].

To address these limitations, considerable research has been devoted to the incorporation of flame retardants (FRs). While halogenated FRs remain prevalent, growing environmental concerns have driven efforts to develop and implement halogen-free alternatives [6,7]. Current strategies in both academic and industrial settings focus on two main categories of flame retardants: additive and reactive types [8]. A key challenge is to ensure that the addition of FRs does not compromise the desirable properties of the foams.

Additive FRs are incorporated via physical mixing and dispersion within the PU matrix [9,10]. However, achieving sufficient flame retardancy often requires large loadings, which can adversely affect foam performance. For instance, effective incorporation of inorganic FRs such as aluminum trihydroxide, magnesium hydroxide, or zinc borate may require loadings exceeding 30 parts per hundred polyol (php), which significantly alters foam morphology and properties [11,12,13,14,15]. Furthermore, additive FRs are prone to migration and leaching during foam use, ultimately diminishing their flame-retardant effectiveness over time [16].

Reactive flame retardants offer a promising solution to these limitations, as they chemically bind to the polymer backbone, ensuring long-term stability and performance. Consequently, increasing research attention has been directed toward the development and application of reactive FR systems [17,18,19].

Viscoelastic polyurethane foams were developed using three halogen-free flame retardants: two additive types-expandable graphite (EG) and ammonium polyphosphate (APP)-and one reactive phosphorus-containing polyol, marketed under the trade name Exolit^®^ OP 550. The aim of this work was to investigate the influence of these flame retardants on the flammability, thermal stability, physico-mechanical properties, and morphology of the foams. Additionally, the potential of the resulting materials was evaluated in the context of developing safer and more sustainable polyurethane products. The findings presented herein may support the design of advanced PU foams for a broad range of industrial applications.

## 2. Materials and Methods

### 2.1. Materials

#### 2.1.1. Substrates for Foam Production

The polyol component (A), characterized by a hydroxyl number (LOH) of 280 mg KOH/g, was prepared according to a proprietary formulation developed by FAMPUR (Bydgoszcz, Poland). This component consists of a mixture of polyols, catalysts, and surfactants, with water added as a chemical blowing agent.

The isocyanate component (B) used was ONGRONAT^®^ TR4040 (BorsodChem, Wanhua, Hungary), a mixture comprising 4,4′-diphenylmethane diisocyanate (MDI), o-(p-isocyanatobenzyl)phenyl isocyanate, and polyisocyanato-polyphenylmethane. This product has a functionality of 2 and contains 32.4 wt.% isocyanate (NCO).

Expandable graphite (EG 290) was supplied by Sinograf (Toruń, Poland). The additive contains a minimum of 90 wt.% carbon and less than 15 wt.% volatile compounds. Its particle size ranges from 0.2 to 0.6 mm, and it exhibits an expansion volume of 200–300 mL/g upon heating.

Exolit^®^ OP 550, obtained from Clariant (Muttenz, Switzerland), is a halogen-free flame retardant specifically designed for use in flexible polyurethane foams. It is a phosphorus-containing reactive polyol with two functional groups and a phosphorus content of 17 wt.%. The acid value of the polyol is approximately 1.8 mg KOH/g, and the hydroxyl number is 170 mg KOH/g. The material is supplied as a viscous liquid, with a viscosity of 3500 mPa·s at 25 °C and a density of 1310 kg/m^3^ at 20 °C.

Exolit^®^ APP 422 (APP 422), also from Clariant (Muttenz, Switzerland), is a fine-grained, non-hygroscopic, non-flammable powder of ammonium polyphosphate (APP). It contains approximately 32 wt.% phosphorus and 15 wt.% nitrogen. The product has a density of 1900 kg/m^3^ and an acid value of less than 1 mg KOH/g. The average particle size of APP 422 is approximately 17 µm.

#### 2.1.2. Foam Synthesis Process

The foams were produced in a single-step process from component A and component B. Additives were added to component A, and after mixing, component B was dosed. The mixture of components A and B was selected in such quantities to achieve an isocyanate index of 0.9. In the calculations of isocyanate content for foams containing Exolit^®^ OP 550, the hydroxyl number of this additive was taken into account.

The mixture was poured into polypropylene containers. After synthesis, the foams were seasoned for 14 days and then cut into test pieces.

The composition of the elastic foams produced is summarized in Table 1.

### 2.2. Methods

#### 2.2.1. Foam Processing Parameters

The synthesis process was evaluated by determining two characteristic times: the cream time (S) and the rise time (W). Cream time was defined as the time elapsed from the mixing of components A and B to the onset of visible volume expansion, while rise time referred to the time required for the mixture to reach its maximum volume. Both parameters were recorded using a digital stopwatch Casio HS-80TW-1EF (Casio, Tokyo, Japan)

#### 2.2.2. Mass Loss Calorimeter (MLC)

The flammability tests were carried out using the cone microcalorimeter MLC from Fire Testing Technology Ltd. (East Grinstead, UK) equipped with a thermocouple. The samples, measuring 100 × 100 × 10 mm, were subjected to a heat flux of 25 kW/m^2^, and the distance from the ignition source was 25 mm. For each foam formulation, three replicates were tested. The following parameters were recorded: peak heat release rate (pHRR), time to pHRR (tpHRR), total heat release (THR), and percent mass loss (PML).

#### 2.2.3. Limiting Oxygen Index (LOI)

The LOI was measured following EN ISO 4589 using an FTT apparatus (Fire Testing Technology Ltd., East Grinstead, UK). The test was conducted at ambient temperature (23 °C), and the minimum oxygen concentration (vol.%) in an O_2_/N_2_ mixture required to sustain combustion was determined with a resolution of 0.1%. Rectangular foam samples with dimensions 10 × 10 × 100 mm were tested, with ten replicates per formulation.

#### 2.2.4. UL-94 Horizontal Flammability Test

Horizontal flammability was evaluated following PN-EN 60695-11-10, equivalent to the UL-94 standard. Tests were carried out in a combustion chamber provided by FTT Sp. z o.o. Foam samples of standard dimensions (125 ± 5 mm × 13 ± 5 mm × 13 mm) were conditioned for 48 h at 23 °C prior to testing. Combustion was initiated using a 50 W gas burner according to IEC 60695-11-4. At least three replicates were tested per foam type. The test classification was based on the following criteria:

- HB40-A—flame does not cross the 25 mm line;
- HB40-B—flame crosses the 25 mm line but the sample does not burn completely;
- HB75—the sample burns completely; burning rate is calculated over a 75 mm segment.

#### 2.2.5. Thermogravimetric Analysis (TGA)

TGA was performed using a Q500 analyzer (TA Instruments, New Castle, DE, USA). Foam samples weighing 10 ± 0.1 mg were placed in platinum crucibles and heated from ambient temperature to 600 °C at a heating rate of 10 °C/min in a nitrogen atmosphere. The data were analyzed using Universal Analysis 2000 software (TA Instruments).

#### 2.2.6. Differential Scanning Calorimetry (DSC)

DSC measurements were conducted using a Q1000 calorimeter (TA Instruments, New Castle, DE, USA). Samples of 6 ± 0.1 mg were placed in sealed aluminum crucibles and analyzed under a helium atmosphere. The heating program included an initial ramp to approximately 200 °C at 10 °C/min, followed by cooling to −90 °C at 5 °C/min, and a second heating run to 200 °C at 10 °C/min. The thermal transitions were analyzed using Universal Analysis 2000 software.

#### 2.2.7. Mechanical Testing—Compression Strength and Comfort Factor

Compression tests were conducted in accordance with EN ISO 3386 using an Instron 5565 universal testing machine (Instron Corporation, Norwood, MA, USA). Foam samples were compressed to 70% of their initial height. Three preconditioning compression-relaxation cycles were applied, and the fourth cycle was used for data acquisition. Foam hardness was defined as the compressive stress at 40% deformation, while additional stress values at 25% and 65% strain were recorded to calculate the SAG comfort factor.

#### 2.2.8. Morphological Analysis (SEM)

The cellular structure of the foams was examined using a Hitachi TM3000 scanning electron microscope (Hitachi Group, Tokyo, Japan). Prior to imaging, samples were sputter-coated with a gold layer using a Polaron SC7640 coater (Quorum Technologies Ltd., Laughton, UK) for 100 s at 7 mA. A minimum of 40 SEM micrographs were taken per sample series. Pore size distribution was quantified using ImageJ software v1.54k.

## 3. Results and Discussion

### 3.1. Results of the Analysis of Characteristic Times of the Foaming Process

The characteristic times of the foaming process are key parameters for describing the kinetics of polyurethane foam formation, providing insights into reaction dynamics and facilitating quality control during production. Table 2 presents the cream time and rise time values recorded during foam synthesis.

The results of the foaming kinetics analysis indicate that the incorporation of flame retardants did not significantly affect the cream time of the polyurethane systems, with the exception of the formulation modified with Exolit^®^ OP 550 (VEF_10OP). However, the addition of flame retardants resulted in an extended rise time in all tested foam systems. The observed increase in both cream and rise times following the incorporation of 10 php Exolit^®^ OP 550 may be attributed to the influence of phosphorus on the foaming kinetics-specifically, its interaction with catalysts involved in the blowing reaction. For the other formulations, the prolonged rise time is likely associated with the increased viscosity of the A-component mixtures due to the presence of solid-phase additives. Higher viscosity limits the mobility of reactive species, thereby slowing down the polymerization reaction and reducing the foam expansion rate. As reported by Członka et al. [20,21], Oliwa et al. [22] and Paciorek-Sadowska et al. [23], the presence of solid additives can disturb the stoichiometry between isocyanate and hydroxyl groups, further affecting the reaction kinetics. Moreover, it is worth noting that expandable graphite is produced through a process involving strong mineral acids, traces of which may remain on the additive surface and interfere with the foaming process, contributing to the observed retardation of foam growth. The introduction of APP into the VEF foam formulation results in an extended rise time compared to the additive-free reference foam. This effect is less pronounced than that observed with the incorporation of an equivalent amount of EG in the VEF_10EG foam. Although both APP and EG are additive powders, they differ significantly in particle size. APP has smaller particles than EG, which leads to a lower viscosity of the A-component mixture with APP compared to the mixture containing EG. As a result, APP imposes a lower restriction on molecular mobility and, consequently, has a less pronounced effect on the polymerization rate and foam expansion in VEF_10APP than EG in VEF_10EG. The addition of EG to the VEF_10APP formulation leads to a slight increase in the cream time and a significant extension of the rise time in the VEF_10APP_20EG and VEF_10APP_30EG foams. The incorporation of EG, a solid additive with relatively large particle size, substantially hinders the mobility of reactive components within the mixture and significantly slows down the foam growth process.

### 3.2. Results of the Analysis of the Physical and Mechanical Properties of Foams

The basic physical and mechanical properties of the foams were characterized and summarized in Table 3.

The results of the analysis indicate an increase in the apparent density of foams (D) after the introduction of flame retardants by 8% to 38%.

The incorporation of OP and EG additives at 10 pphp results in only minor changes in the apparent density of the foams. The VEF_10APP foam exhibits a significantly higher apparent density compared to VEF_10EG and VEF_10OP foams. Among all the flame retardants used, APP contributes to the highest density increase.

The greatest increase in foam density was obtained for materials with the highest content of flame retardants (VEF_10OP_30EG and VEF_10APP_30EG).

The change in apparent density of the foams containing additives results from modifications in the cellular structure induced by the incorporation of reactive Exolit^®^ OP 550 into the initial system. A second contributing factor is the use of solid-phase additives -EG and APP, whose intrinsic densities are significantly higher than the ap-parent density of the polyurethane matrix.

In the context of furniture manufacturing, user comfort is a critical parameter, often quantified by the comfort factor, also known as the SAG factor. The results indicate favorable SAG values exceeding 2 for all tested formulations. The incorporation of flame retardants generally led to an increase in the SAG factor, which may be advantageous for the potential application of modified foams in comfort-sensitive products. A slight decrease in the SAG factor, in the range of 3–6%, was observed only for the VEF_10OP and VEF_10APP formulations.

Foam hardness was evaluated as the compressive stress at 40% deformation (H40%) of the sample height. This parameter is frequently correlated with both the foam’s apparent density and its chemical structure.

For the materials under investigation, no consistent correlation was observed. The hardness test results indicate a significant influence of both the type and the amount of flame retardant (FR) on this property. The incorporation of 10 pphp EG into the PU matrix (VEF_10EG) resulted in an increase in both apparent density and hardness. However, further addition of EG at 20 and 30 pphp led to a decrease in hardness compared to VEF_10EG, despite the continued increase in apparent density.

The use of phosphorus-based polyol Exolit^®^ OP 550 caused a reduction in the hardness of the foams (VEF_10EG, VEF_10OP_20EG), despite the increase in their density. This suggests a plasticizing effect of Exolit^®^ OP 550, even when combined with an additional 20 pphp of EG. Increasing the EG content to 30 pphp in this series resulted in a notable increase in foam hardness. The explanation for this phenomenon will be discussed in the following sections, based on the analysis of the cellular and phase structure of the foams.

The incorporation of 10 pphp APP into the VEF foam led to an increase in both apparent density and hardness, with the hardness of the VEF_10APP sample increasing by approximately 52%. The addition of 20 pphp EG to this formulation resulted in a decrease in hardness of about 27% compared to VEF_10APP. Increasing the EG content to 30 pphp in the VEF_10APP_30EG foam caused an increase in hardness by approximately 27% compared to VEF_10APP_20EG. The mechanisms responsible for these changes require further investigation.

### 3.3. Flammability Analysis

The values of key combustion parameters-including peak heat release rate (pHRR), time to pHRR (tpHRR), total heat release (THR), and percentage mass loss (PML)-obtained from cone microcalorimetry tests are summarized in Table 4. In addition to these indicators, the fire growth rate index (FIGRA) was also calculated to assess the potential rate of fire development. FIGRA is defined as the ratio of pHRR to tpHRR and is increasingly used as a predictive parameter for estimating fire hazard levels. Lower FIGRA values correspond to slower flame spread, thereby offering more time for safe evacuation and fire suppression.

**Figure 1:**
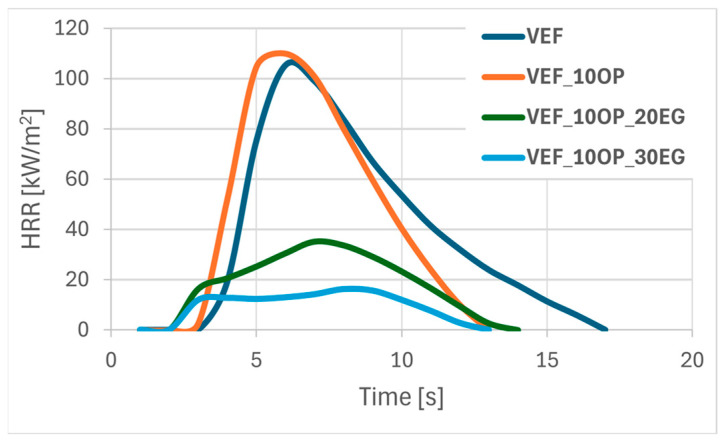
Summary of HRR curves for VEF, VEF_10OP, VEF_10OP_20EG, and VEF_10OP_30EG materials.

**Figure 2:**
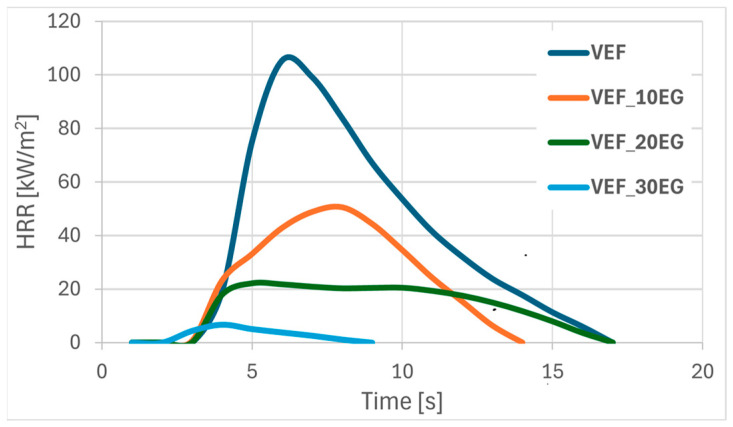
Summary of HRR curves for VEF, VEF_10EG, VEF_20EG, and VEF_30EG materials.

**Figure 3:**
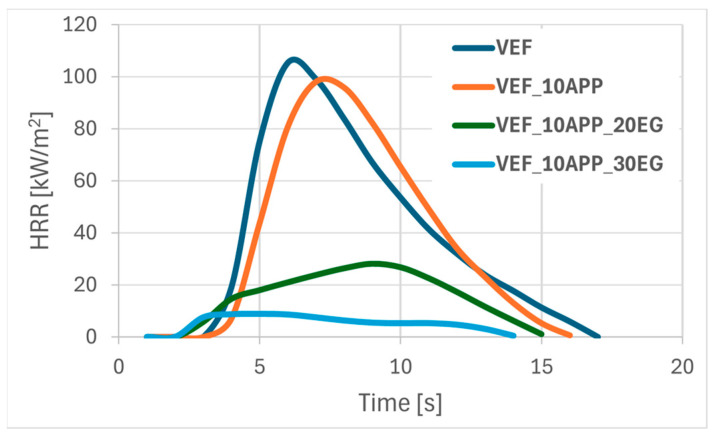
Summary of HRR curves for materials VEF, VEF_10APP, VEF_10APP_20EG, VEF_10APP_30EG.

**Figure 4:**
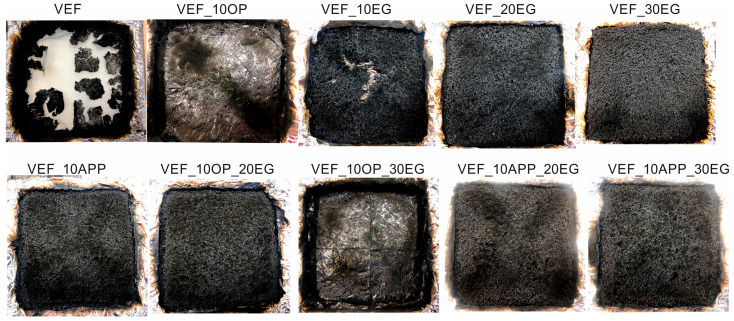
Foams look after flammability tests.

Figure 1, Figure 2 and Figure 3 present representative heat release rate (HRR) curves as a function of time for selected foam formulations. Post-pyrolysis and combustion residues are shown in Figure 4. For all tested materials, limiting oxygen index (LOI) measurements were also performed, and the flammability classification was determined according to the UL-94 standard.

The results of the flammability analysis conducted using cone calorimetry revealed that the incorporation of the phosphorus-containing polyol Exolit^®^ OP 550 into the reference VEF system led to a slight 2% increase in the peak heat release rate (pHRR) and a reduction in the time to reach pHRR (tpHRR), ultimately resulting in an increase in the FIGRA index. The HRR profiles for VEF and VEF_10OP samples were found to be similar (Figure 1). A beneficial reduction in total heat release (THR) and an increase in the limiting oxygen index (LOI) were observed. However, based on literature criteria, the obtained LOI value classifies the VEF_10OP sample as a flammable plastic, as the threshold LOI for self-sustaining combustion is typically above 21% (Table 4) [24]. This low flame resistance is further corroborated by the UL-94 horizontal test results, in which the material burned completely.

The incorporation of Exolit^®^ OP 550 did not significantly influence the residue content after combustion, but it did affect char morphology. The char layer formed on the surface of the unmodified VEF foam appeared heterogeneous and cracked, while a continuous, more stable char structure was observed in the VEF_10OP foam (Figure 4).

Modification of the VEF reference system with expandable graphite (EG) resulted in a substantial reduction in pHRR by 52%, 88%, and 92% for foams containing 10, 20, and 30 php of EG, respectively (Figure 2, Table 4). While the HRR curve for VEF_10EG remained similar to that of the reference foam, the HRR curves for the higher EG loadings flattened noticeably, indicating more controlled combustion behavior. Incorporating 10 php EG improved tpHRR, but higher EG loadings resulted in its reduction. Additionally, a consistent decrease in THR was observed with increasing EG content, likely due to the formation of an insulating, swollen graphite char layer that restricted oxygen access and flame propagation to the inner foam structure. This is supported by the increase in post-combustion residue from 9% in the reference sample to 45% in the VEF_30EG formulation. The improved fire resistance was further reflected in the significantly lower FIGRA values.

Char analysis following cone calorimetry showed a typical ‘snail-like’ structure of expanded graphite on the surface of VEF_10EG, VEF_20EG, and VEF_30EG samples. Cracks in the char layer were visible in VEF_10EG due to internal pressure buildup during combustion, while more coherent char layers were observed for the 20 and 30 php EG formulations. The 30 php EG foam also demonstrated improved fire behavior, achieving a V_0_ flammability rating in the UL-94 test and a significantly increased LOI.

The combined use of 10 php Exolit^®^ OP 550 with 20 or 30 php of EG in VEF_10OP_20EG and VEF_10OP_30EG formulations yielded less effective reductions in pHRR-69% and 89%, respectively-compared to systems containing only EG (VEF_20EG and VEF_30EG). However, a significant advantage was observed in the extension of tpHRR, increasing from 12 s (VEF_30EG) to 42 s (VEF_10OP_30EG). This delay translated into a reduced FIGRA value, indicating improved fire safety. The VEF_10OP_30EG foam also achieved a V0 rating in the UL-94 test and an LOI of 28.2%. The morphology of the char formed in these samples resembled that observed in EG-modified foams, suggesting that the graphite still dominated the char formation process.

Substituting Exolit^®^ OP 550 with ammonium polyphosphate (APP) in flame retardant systems containing EG yielded the most pronounced improvement in flammability performance (Table 4, Figure 3). The VEF_10APP_30EG formulation exhibited the lowest THR, the longest tpHRR, and a remarkably low pHRR of 9 kW/m^2^, resulting in an eightfold reduction in FIGRA relative to the reference foam. Materials containing both APP and EG achieved a V_0_ flammability classification in UL-94 tests and exhibited high LOI values in the range of 28–30%.

Both EG and APP exhibit intumescent behavior upon heating. The use of these additives enables the formation of a continuous and stable char layer on the surface of the foams during combustion, resulting in a synergistic flame-retardant effect (Figure 4).

The modification of graphite-based flame retardant systems through the substitution of the phosphorus-containing polyol Exolit^®^ OP 550 with APP powder not only enhances flame retardancy but also offers economic advantages. Unlike Exolit^®^ OP 550, the use of APP does not require additional isocyanate, thereby reducing material costs and improving the overall cost-effectiveness of the formulation.

### 3.4. Thermal Analysis

**Figure 5:**
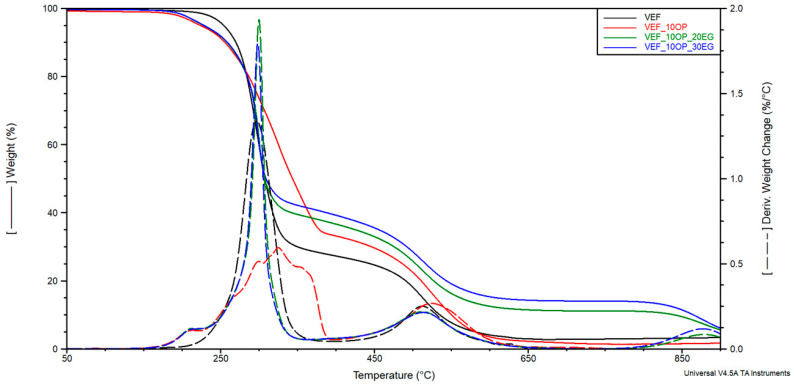
TG and DTG curves for VEF, VEF_10OP, VEF_10OP_20EG, and VEF_10OP_30EG materials.

**Figure 6:**
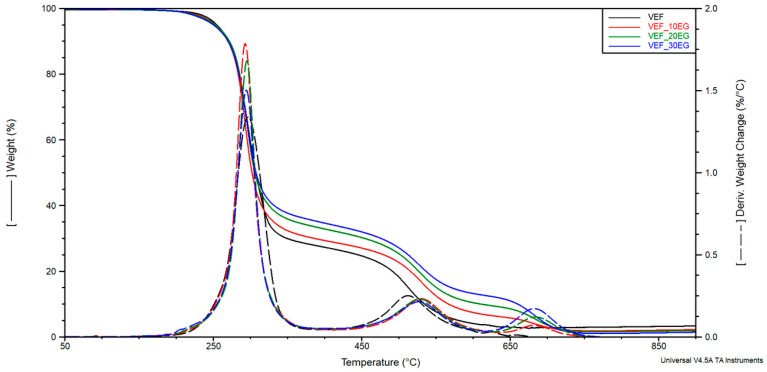
TG and DTG curves for VEF, VEF_10EG, VEF_20EG, and VEF_30EG materials.

**Figure 7:**
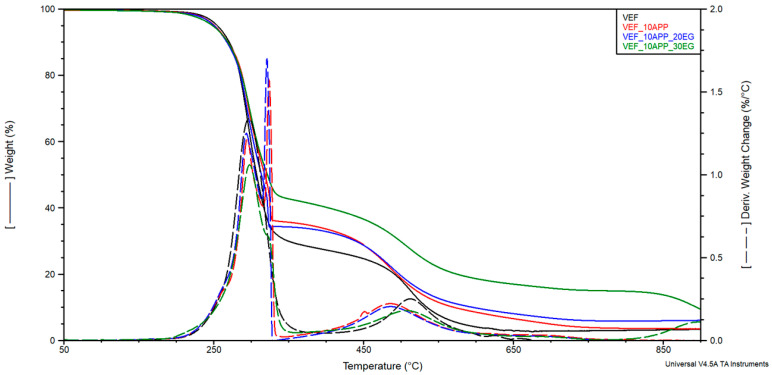
TG and DTG curves for VEF, VEF_10APP, VEF_10APP_20EG, and VEF_10APP_30EG materials.

Based on the curves of mass change as a function of temperature (TG), the temperature of 2% mass loss (T_2%_) and the residue at 800 °C (R_800_) were determined, as well as the mass changes Δm_1_, Δm_2_, Δm_3_ corresponding to the successive stages of material degradation in the temperature ranges of 200–400 °C, 400–630 °C and 630–750 °C. The curves of the derivative of mass change as a function of temperature (DTG) were used to determine the maximum degradation rates Vmax in successive stages of degradation and, respectively, the temperatures T_max_ at which these rates were achieved. The curves of mass change (TG) and derivative of mass change (DTG) are shown in Figure 5, Figure 6 and Figure 7. The results are summarized in Table 5.

The thermal degradation of the reference viscoelastic foam (VEF) proceeds in two distinct stages. The first degradation stage is characterized by a maximum weight loss rate (V_max1_) of 1.45%/°C at 295 °C, with a corresponding mass loss (Δm_1_) of approximately 72%. This stage is primarily attributed to the thermal decomposition of urea and urethane linkages present in the hard segments, as well as the initial degradation of soft segments, in agreement with previous studies [22,25,26,27,28].

The second degradation stage occurs at 512 °C, with a lower maximum degradation rate (V_max2_) of 0.26%/°C and a mass loss (Δm_2_) of about 24%. This phase corresponds to the decomposition of remaining flexible segments and the pyrolysis of residual char from the first stage.

Modification of the reference system with the phosphorus-containing polyol Exolit^®^ OP 550 led to a reduction in mass loss during the first degradation stage and a corresponding increase in Δm_2_ in the second stage. This shift suggests improved thermal stability in the early stages of degradation. In formulations containing expandable graphite (EG), a progressive reduction in Δm_1_ was observed with increasing EG content. This behavior confirms the formation of a protective insulating layer that limits thermal degradation and flame spread-an effect also visually confirmed through post-combustion residue images obtained after cone calorimetry testing (Figure 4).

In systems modified with EG, a third degradation stage was identified on the DTG curves, corresponding to the decomposition of char residues in the 630–750 °C temperature range. The maximum degradation temperature for this third stage (T_max3_) was observed between 682–684 °C. The use of flame retardant systems combining EG with either Exolit^®^ OP 550 or APP resulted in a similar reduction in material decomposition during the first stage. This can be attributed to the synergistic effect of the insulating char layer formed by EG and the flame-retardant action of the phosphorus-based additives, which together limited heat transfer into the foam structure.

Moreover, these modified systems exhibited higher residual mass at 800 °C, indicating enhanced char stability. The temperature corresponding to 2% mass loss was found to decrease upon flame retardant addition, suggesting a higher content of volatile compounds in the modified formulations.

**Figure 8:**
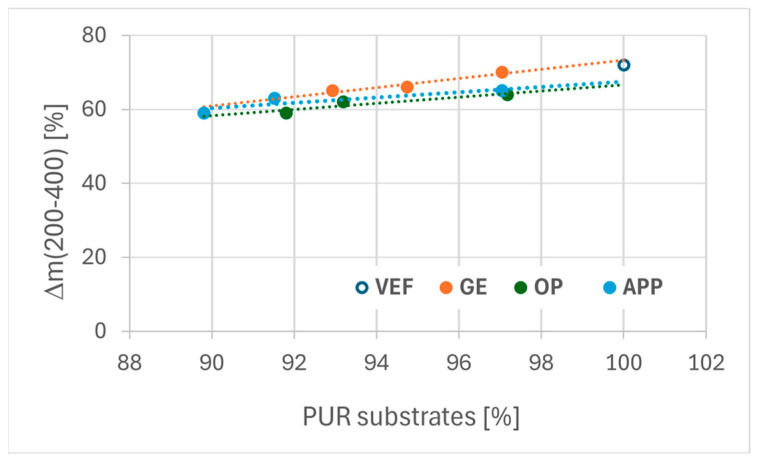
Analysis of the relationship between the change in the proportion of PUR-forming substrates in the foam and the change in weight in the first stage of degradation in the range of 200–400 °C.

An analysis of PUR substrate composition and weight change during the first degradation stage revealed that mass loss during this phase correlates with the content of polyurethane-forming components. This relationship is presented in Figure 8. Notably, foams modified with Exolit^®^ OP 550 displayed lower mass loss in the initial degradation stage compared to EG- and APP-modified series, indicating that Exolit^®^ OP 550 effectively hinders PUR decomposition in the early stages of thermal degradation.

Based on differential scanning calorimetry (DSC) thermograms, the glass transition temperature (Tg) and the specific heat capacity change (Δcp) associated with the glass transition were determined for all tested foam formulations. The reference VEF foam exhibited a Tg of approximately −6.5 °C and a Δcp value of 0.15 J/g·°C.

The incorporation of the reactive phosphorus-based polyol Exolit^®^ OP 550 in the VEF_10OP foam resulted in a significant reduction in Tg to approximately −18 °C, accompanied by a more than threefold increase in Δcp to ~0.51 J/g·°C. This substantial change suggests a notable enhancement in the flexibility of the polymer chains, confirming the plasticizing effect of Exolit^®^ OP 550.

The introduction of expandable graphite (EG) into the VEF formulation led to a slight increase in Tg and a marked increase in Δcp. This behavior suggests that EG facilitates the segmental mobility of polymer chains within the temperature range associated with the glass transition.

In systems containing both Exolit^®^ OP 550 and EG, a significant increase in Tg and a decrease in Δcp were observed in comparison to VEF_10OP. These results indicate that EG counteracts the plasticizing effect of Exolit^®^ OP 550, likely due to interactions between the solid additive and the polymer matrix, which restrict molecular mobility.

A similar trend was observed in foams modified with Exolit^®^ APP 422 (ammonium polyphosphate). The addition of APP to the VEF foam led to plasticization, evidenced by a Tg decrease to −14 °C and an increase in Δcp to 0.47 J/g·°C. However, the subsequent incorporation of EG into the VEF_10APP formulation significantly mitigated the plasticizing effect, resulting in an increase in Tg and a reduction in Δcp.

**Figure 9:**
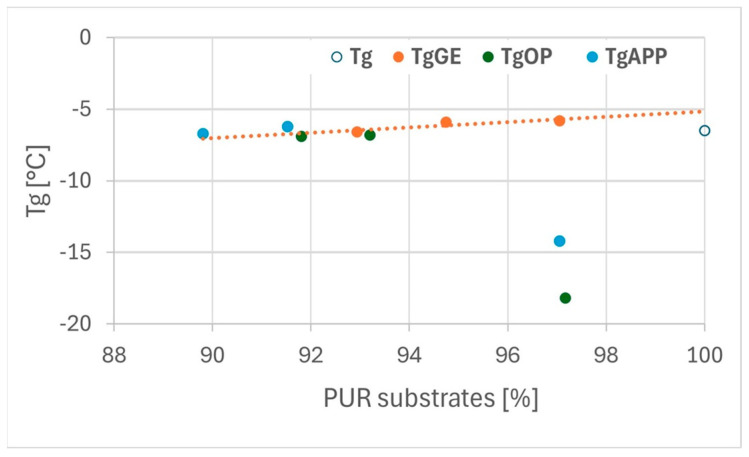
The relationship between the Tg of foams and the content of PUR-forming substrates in these foams: Tg—glass transition temperature of VEF foam; Tg EG, Tg OP, Tg APP—glass transition temperature of series with expanding graphite, Exolit^®^ OP 550, Exolit.

**Figure 10:**
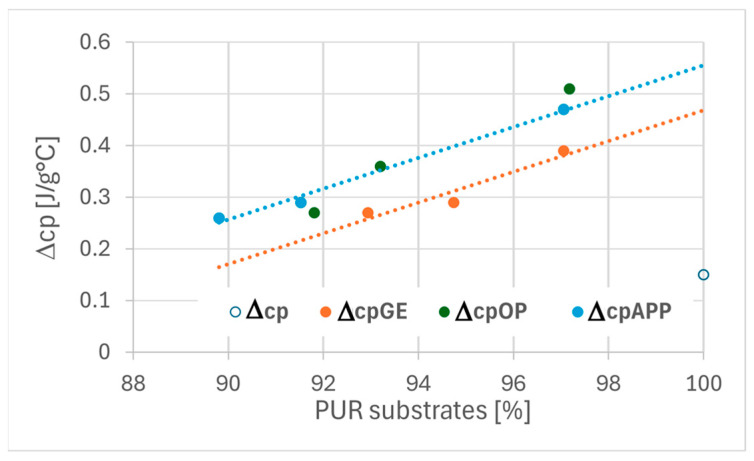
The relationship between the specific heat (Δcp) of foams and the content of PUR substrates in these foams: Δcp- specific heat of VEF foam; Δcp EG, Δcp OP, Δcp APP- specific heat of series with expanding graphite, Exolit^®^ OP 550, Exolit^®^ APP 420, respectively.

The correlation between polyurethane substrate composition and the thermal transition parameters (Tg and Δcp) is illustrated Figure 9 and Figure 10. These results demonstrate that both the chemical structure and the composition of flame retardant systems play a crucial role in defining the thermal behavior and molecular mobility of viscoelastic polyurethane foams.

As shown in Figure 9, the majority of the tested foams exhibit similar glass transition temperatures (Tg), with the exception of VEF_10OP and VEF_10APP formulations, which display significantly lower Tg values. This observation confirms that the addition of Exolit^®^ OP 550 and Exolit^®^ APP 422 imparts a plasticizing effect on the VEF foam matrix, enhancing the flexibility of the polymer chains.

The analysis of the specific heat change (Δcp) at Tg (Figure 10) reveals a positive correlation between the content of polyurethane-forming substrates and the specific heat capacity in all tested foams. Notably, foams modified with expandable graphite exhibit slightly lower Δcp values compared to those containing phosphorus-based flame retardants. This suggests reduced molecular mobility in the segmental domains associated with the glass transition in EG-containing foams.

The reduced segmental mobility or increased stiffness of the soft phase in EG-modified foams may be attributed to structural changes within the hard phase. Literature reports indicate that rigid segments in polyurethane systems can chemically interact with expandable graphite, thereby limiting the flexibility of the polymer network [29,30].

### 3.5. Microstructure Analysis

**Figure 11:**
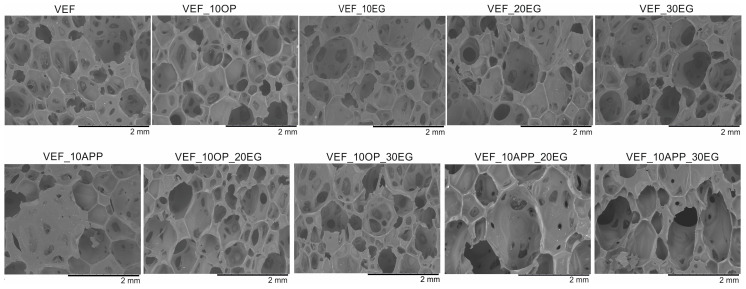
Images of foams microstructure at 40× magnification.

**Figure 12:**
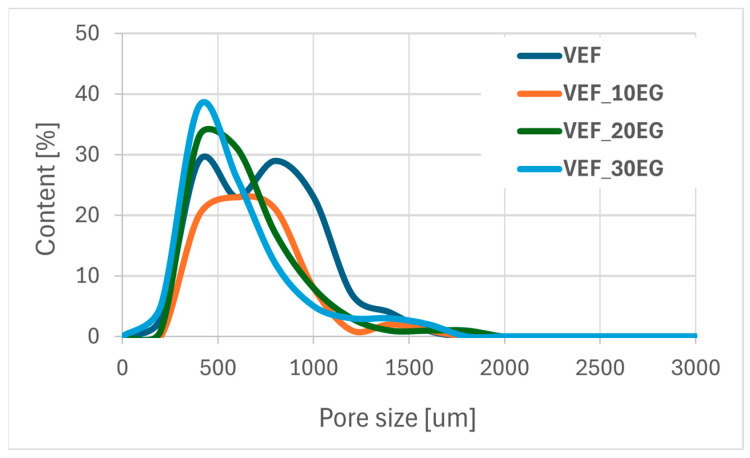
Pore size distribution of a series of foams with expanded graphite.

**Figure 13:**
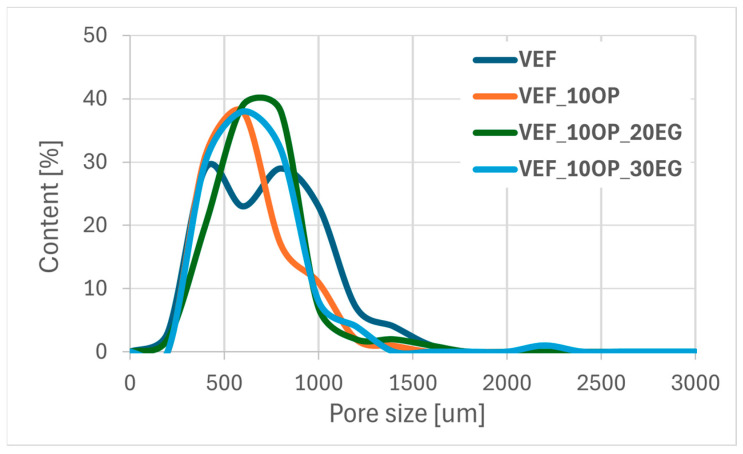
Pore size distribution of foam series with Exolit^®^ OP 550.

**Figure 14:**
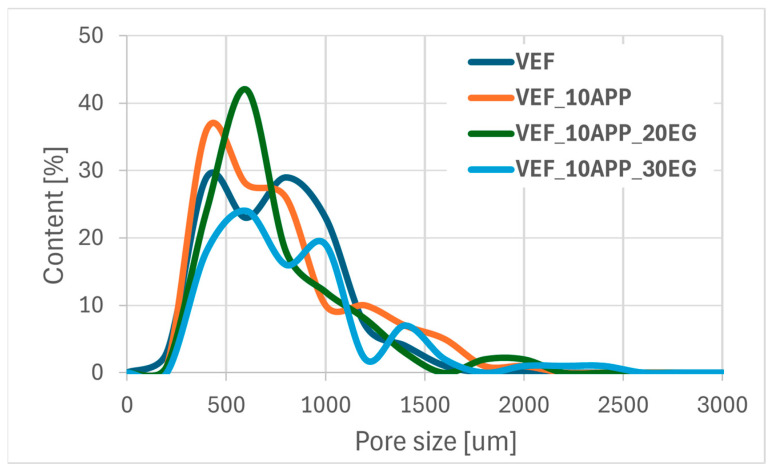
Pore size distribution of foam series with Exolit^®^ APP 422.

Using scanning electron microscopy, the microstructure of the foams was described (Figure 11), the pore sizes were determined (Table 6) and their size distribution was determined (Figure 12, Figure 13 and Figure 14).

The reference VEF foam is characterized by a high proportion of oval-shaped pores, with cell sizes ranging from 140 to 1430 µm and an average equivalent diameter of approximately 700 µm. The incorporation of the phosphorus-containing polyol Exolit^®^ OP 550 led to the formation of pores with more regular geometry and a reduction in the average equivalent diameter.

In contrast, the addition of expandable graphite (EG) into the VEF matrix increased the overall range of pore sizes. As the EG content increased, the minimum pore diameter decreased, although the average equivalent diameter remained relatively constant at approximately 600 µm, regardless of the EG concentration.

In foams containing both EG and Exolit^®^ OP 550, the cellular structure exhibited less regular pore shapes and broader pore size distributions. Nevertheless, the average equivalent pore diameter remained similar to that of the EG-only series, at approximately 580 µm.

The combined use of ammonium polyphosphate (APP) and EG in the VEF system resulted in both an increase in average pore diameter and an expansion of the overall pore size distribution. This significant enlargement of the cellular structure is attributed to the presence of two solid-phase flame retardants, which increase the viscosity of the reacting system. The elevated viscosity restricts bubble coalescence and drainage dynamics during foaming, leading to altered cell morphology-a phenomenon consistent with findings reported by other authors [27].

An increase in the content of expandable graphite (EG) in the VEF foam formulation leads to a decrease in the proportion of pores with diameters below 700 µm, accompanied by an increase in the number of larger pores.

In the Exolit^®^ OP 550 series, the addition of EG to the VEF_10OP formulation results in a higher proportion of pores with diameters up to 620 µm, indicating a shift toward smaller and more uniform cell structures in the system.

Conversely, the incorporation of both Exolit^®^ APP 422 and EG into the VEF matrix promotes the formation of larger pores, particularly those exceeding 800 µm. This suggests that the presence of solid-phase flame retardants facilitates pore coalescence and bonding, likely due to their influence on system viscosity and cell wall stability during the foaming process.

## 4. Discussion and Conclusions

The goal was to identify a flame-retardant system capable of minimizing the flammability of viscoelastic polyurethane foams (VEFs). To this end, foams modified with a reactive phosphorus-based polyol (Exolit^®^ OP 550) and two solid additive flame retardants-expandable graphite (EG) and ammonium polyphosphate (APP, Exolit^®^ APP 422)-were prepared and evaluated.

Exolit^®^ OP 550 was introduced as a liquid reactive flame retardant at a concentration of 10 pphp. For comparative analysis, additional formulations were prepared by incorporating 20 and 30 pphp of EG into the VEF_10OP system. Similarly, a parallel series was developed using APP, in which 10 pphp APP was introduced into the reference foam, followed by the addition of 20 and 30 pphp EG into the VEF_10APP matrix.

Flammability tests revealed that the VEF_10OP_30EG and VEF_10APP_30EG foams exhibited the most favorable fire safety profiles. These materials achieved the lowest fire growth rate index (FIGRA) values of 0.3 and 0.5 kW/m^2^s, respectively, and low peak heat release rates (pHRR) of 12 and 9 kW/m^2^. Additionally, both foams demonstrated limiting oxygen index (LOI) values exceeding 28% and achieved a V-0 classification in the UL-94 flammability test.

The combustion behavior of these foams was consistent with intumescent materials. A coherent and stable char layer formed on the surface during combustion, acting as a physical barrier to heat and mass transfer and contributing to the enhanced fire resistance.

Slightly higher fire hazard was observed for the VEF_30EG and VEF_10APP_20EG foams. These materials achieved a V-0 flammability classification, with FIGRA values of 0.8 kW/m^2^s and 0.7 kW/m^2^s, respectively, and limiting oxygen indices (LOI) of 26.3% and 28.0%, respectively. A significant reduction in flammability was recorded for the foams VEF_30EG, VEF_10OP_30EG, VEF_10APP_20EG, and VEF_10APP_30EG, with the enhanced fire resistance in each case being attributed to different combustion mechanisms.

In the case of the VEF_30EG foam, during combustion, EG undergoes intumescence and forms a protective char layer on the foam surface. This barrier limits heat transfer and gas exchange, effectively lowering the fire hazard. Moreover, the expansion of EG is an endothermic process that absorbs heat, further enhancing the flame resistance of the material.

To improve the flame retardancy of VEF_30EG, the reactive phosphorus-based polyol Exolit^®^ OP 550 was introduced into the formulation. In the VEF_10OP_30EG foam, improved fire performance results from a synergistic effect between EG and OP. The char layer formed by expanding EG limits heat and gas exchange, while the chemical incorporation of phosphorus-containing structures into the polyurethane macro-molecule increases the material’s thermal resistance. The combined effect of both mechanisms leads to a substantial reduction in fire hazard.

The VEF_10APP_20EG and VEF_10APP_30EG foams also contain EG, whose mechanism of action has been described above. In these formulations, a synergistic effect is additionally obtained by the inclusion of Exolit^®^ APP 422, which is an ammonium polyphosphate in the crystalline form known as phase II (APP II). This type of APP decomposes at approximately 300 °C into polymeric phosphoric acid and ammonia. The polymeric acid acts as a catalyst for the formation of a protective char layer. During heating, APP II absorbs heat through processes such as softening, melting, evaporation, and char formation, which contributes to reduced fire hazard. The char formed from both EG and APP II provides effective thermal insulation and limits heat transfer, which accounts for the low flammability of these foams.

After degradation of these foams in nitrogen at 800 °C, 1–15% of ash remained. The weight loss in the first stage of foam degradation decreases with the decrease in the proportion of polyurethane components. DSC analysis showed that the glass transition temperature of the soft phase of VEF_10OP and VEF_10APP foams is significantly lower than that of the reference VEF foam, indicating that both additives plasticize polyurethane. The glass transition temperature of the soft phase of the other foams does not change with the change in the proportion of polyurethane substrates in these foams. The change in specific heat at the glass transition temperature of the foams is the result of a change in phase ordering. Expanding graphite bonds with segments of the hard phase of the foams, resulting in increased mobility of the soft phase.

As a result of pore microstructure analysis, it was found that in VEF_10OP_30EG, VEF_10APP_20EG and VEF_10APP_30EG foams, pores with the highest average pore diameter were formed, and the size of these pores does not affect the flammability of these foams.

The produced foams exhibit the physical and mechanical properties characteristic of typical viscoelastic foams. In the series containing only EG, an increase in the EG content results in higher apparent density but reduced hardness. The decrease in hardness is attributed to enhanced mobility of soft segments within the macromolecular chains, caused by chemical interactions between the expandable graphite (EG) and the hard segments of the foam.

In the series containing Exolit^®^ OP 550 and Exolit^®^ APP 422, the incorporation of 20 pphp EG into the VEF_10OP_20EG and VEF_10APP_20EG foams leads to a notable reduction in hardness. This softening effect may also be related to interactions between EG and the hard segments of the polyurethane matrix.

Further increase in EG content to 30 pphp in both series results in a significant in-crease in hardness-approximately 70% for VEF_10OP_30EG and 45% for VEF_10APP_30EG-compared to their respective 20 pphp EG counterparts. This phenomenon may be due to the limited capacity for additional EG to chemically interact with hard segments at higher concentrations. Consequently, the mobility of soft segments is slightly reduced, and EG particles begin to act as physical barriers to the motion of both soft and hard segments within the macromolecular structure, ultimately leading to increased foam hardness.

The effectiveness of the reactive flame retardant additive Exolit^®^ OP 550 is lower than that of the additive Exolit^®^ APP 422. Furthermore, the use of a reactive additive requires the use of a larger amount of isocyanate, one of the more expensive PUR substrates.

The results presented may be a valuable source of information for both scientists and entrepreneurs looking for effective solutions in the development of safe-to-use products.

## Figures and Tables

**Table 1:** Composition of the tested foams and synthesis process parameters.

	Additives in Polyols, php			Additives in Foam Substrates, wt. %			
Sample	OP 550	EG 290	APP 422	OP 550	EG 290	APP 422	PU Substrates
VEF	0	0	0	0	0	0	100.0
VEF_10OP	10	0	0	5.8	0	0	97.2
VEF_10EG	0	10	0	0	6.0	0	97.1
VEF_20EG	0	20	0	0	11.2	0	94.7
VEF_30EG	0	30	0	0	16.0	0	92.9
VEF_10OP_20EG	10	20	0	5.2	10.4	0	93.2
VEF_10OP_30EG	10	30	0	5.0	14.9	0	91.8
VEF_10APP	0	0	10	0	0	6.0	97.1
VEF_10APP_20EG	0	20	10	0	10.6	5.3	91.5
VEF_10APP_30EG	0	30	10	0	15.1	5.1	89.9

**Table 2:** Characteristic foaming reaction times of PUR.

Sample	Start Time, s	Rise Time, s
VEF	12	257
VEF_10OP	17	320
VEF_10EG	10	407
VEF_20EG	11	409
VEF_30EG	12	430
VEF_10OP_20EG	12	404
VEF_10OP_30EG	12	406
VEF_10APP	12	330
VEF_10APP_20EG	13	441
VEF_10APP_30EG	13	463

**Table 3:** Physicomechanical specimen properties.

Sample	D [kg/m^3^]	SAG Factor	H40% [kPa]
VEF	36.16 ± 0.56	2.58 ± 0.04	2.58 ± 0.31
VEF_10OP	39.39 ± 1.21	2.50 ± 0.07	2.32 ± 0.02
VEF_10EG	39.22 ± 0.51	2.66 ± 0.11	2.72 ± 0.11
VEF_20EG	41.23 ± 0.58	2.88 ± 0.06	2.69 ± 0.04
VEF_30EG	45.00 ± 0.14	3.07 ± 0.12	2.54 ± 0.05
VEF_10OP_20EG	47.09 ± 1.33	3.24 ± 0.06	1.91 ± 0.11
VEF_10OP_30EG	50.02 ± 1.01	3.45 ± 0.49	3.24 ± 0.21
VEF_10APP	42.68 ± 1.50	2.42 ± 0.08	3.92 ± 0.01
VEF_10APP_20EG	44.84 ± 1.62	2.99 ± 0.03	2.85 ± 0.06
VEF_10APP_30EG	48.87 ± 2.13	3.16 ± 0.06	3.93 ± 0.12

**Table 4:** Flame tests results.

Sample	pHRR kW/m^2^	tpHRR s	THR MJ/m^2^	PML %	FIGRA kW/m^2^s^1^	LOI %	UL94
VEF	109 ± 6	28 ± 4	5.4 ± 0.1	91 ± 1	4.0 ± 0.3	17.8	HB40
VEF_10OP	111 ± 6	23 ± 3	4.3 ± 0.6	90 ± 1	4.8 ± 0.5	20.9	NC *
VEF_10EG	53 ± 9	35 ± 0	4.4 ± 0.5	81 ± 4	1.5 ± 0.3	21.4	HB40
VEF_20EG	21 ± 5	18 ± 3	4.2 ± 1.0	63 ± 4	1.1 ± 0.1	24.3	HB40
VEF_30EG	9 ± 3	12 ± 3	2.8 ± 0.1	55 ± 5	0.8 ± 0.4	26.3	V-0
VEF_10OP_20EG	34 ± 3	33 ± 6	4.2 ± 0.5	72 ± 2	1.0 ± 0.1	26.3	HB40
VEF_10OP_30EG	12 ± 6	42 ± 6	3.6 ± 0.2	61 ± 3	0.3 ± 0.2	28.2	V-0
VEF_10APP	93 ± 9	30 ± 5	3.4 ± 0.5	92 ± 1	3.1 ± 0.3	20.6	NC *
VEF_10APP_20EG	29 ± 2	42 ± 3	3.6 ± 0.1	70 ± 2	0.7 ± 0.0	28.0	V-0
VEF_10APP_30EG	9 ± 0	18 ± 4	2.7 ± 0.3	55 ± 2	0.5 ± 0.1	30.6	V-0

* The sample cannot be classified using this method; the material burned completely.

**Table 5:** Results of TG, DTG, and DSC thermogram analysis.

	T_2%_ [°C]	T_max1_ [°C]/ V_max1_ [%/°C]	Δm_(200–400)_, %	T_max2_ [°C]/ V_max2_ [%/°C]	Δm _(400–630)_, %	T_max3_ [°C]/ V_max2_ [%/°C]	Δm _(630–750)_, %	R_800_, %	Tg [°C]	Δcp [J/g·°C]
VEF	234	295/1.45	72	512/0.26	24	-	0.3	3,0	−6.5	0.15
VEF_10OP	193	325/0.59	64	526/0.27	31	-	1.1	1,4	−18.2	0.51
VEF_10EG	227	292/1.78	70	530/0.23	23	682/0.07	4.4	1.9	−5.8	0.39
VEF_20EG	225	295/1.68	66	528/0.23	24	684/0.12	7.7	1.6	−5.9	0.29
VEF_30EG	222	294/1.50	65	527/0.21	23	683/0.17	11.0	1.1	−6.6	0,27
VEF_10OP_20EG	202	300/1.93	62	512/0.22	25	-	0.5	11	−6.8	0.36
VEF_10OP_30EG	202	298/1.80	59	514/0.21	25	-	0.5	13.8	−6.9	0.27
VEF_10APP	229	295/1.22 324/1.57	65	486/0.22	26	-	3.4	3.6	−14.2	0.47
VEF_10APP_20EG	222	297/1.17	63	507/0.19	24	-	3.1	9.9	−6.2	0.29
VEF_10APP_30EG	221	298/1.06	59	508/0.18	23	-	2.4	14.8	−6.7	0.26

**Table 6:** Pore analysis table.

Sample	Size Intervals, μm	Average Pore Sizes, μm	Size Intervals, μm
VEF	140–1430	696	636
VEF_10OP	220–1260	573	518
VEF_10EG	215–1460	634	628
VEF_20EG	190–1760	606	665
VEF_30EG	100–1450	595	670
VEF_10OP_20EG	140–1550	621	582
VEF_10OP_30EG VEF_10APP	120–1040 180–1850	613 793	659 940
VEF_10APP_20EG	160–1960	721	809
VEF_10APP_30EG	200–2270	831	920

## Data Availability

The original contributions presented in the study are included in the article, further inquiries can be directed to the corresponding author.

## Abbreviations

The following abbreviations are used:

VEF	Viscoelastic foam
LOI	limiting oxygen index
UL94	Flammability test
pHRR	Peak heat release rate
tpHRR	Time to reach pHRR
THR	Total heat release
PML	Percentage mass loss
FIGRA	Fire growth rate index
SEM	Scanning electron microscopy
TGA	Thermogravimetric analysis
TG	Curve of mass change as a function of temperature
DTG	Curve of the derivative of mass change as a function of temperature
T2%	Temperature of 2% mass loss
R800	Residue at 800 °C
Δm	Change in mass at a specific stage of degradation
V_max_	Maximum degradation rate at a specific stage of degradation
T_max_	Temperature at which maximum degradation rate were achieved
DSC	Differential scanning calorimetry
Tg	Glass transition temperature
Dcp	Specific heat in the scope of glass transition transformation
PU	Polyurethane
FR	Flame retardant
php	Per hundred polyol
LOH	Hydroxyl number
EG	Expandable graphite
OP 550	Exolit^®^ OP 550
APP 422	Exolit^®^ APP 422
